# Microfluidics-based side view flow chamber reveals tether-to-sling transition in rolling neutrophils

**DOI:** 10.1038/srep28870

**Published:** 2016-06-30

**Authors:** Alex Marki, Edgar Gutierrez, Zbigniew Mikulski, Alex Groisman, Klaus Ley

**Affiliations:** 1 La Jolla Institute for Allergy and Immunology, La Jolla, CA, USA; 2 Department of Physics and University of California San Diego, La Jolla, CA, USA; 3 Department of Bioengineering, University of California San Diego, La Jolla, CA, USA.

## Abstract

Neutrophils rolling at high shear stress (above 6 dyn/cm^2^) form tethers in the rear and slings in the front. Here, we developed a novel photo-lithographically fabricated, silicone(PDMS)-based side-view flow chamber to dynamically visualize tether and sling formation. Fluorescently membrane-labeled mouse neutrophils rolled on P-selectin substrate at 10 dyn/cm^2^. Most rolling cells formed 5 tethers that were 2–30 μm long. Breaking of a single tether caused a reproducible forward microjump of the cell, showing that the tether was load-bearing. About 15% of all tether-breaking events resulted in slings. The tether-to-sling transition was fast (<100 ms) with no visible material extending above the rolling cell, suggesting a very low bending modulus of the tether. The sling downstream of the rolling cell aligned according to the streamlines before landing on the flow chamber. These new observations explain how slings form from tethers and provide insight into their biomechanical properties.

Neutrophil granulocytes are the first responder immune cells in bacterial and fungal infections. Most of their immune response activity occurs outside of the blood vessels, requiring extravasation^1^,^2^,^3^. As a first step of extravasation, streaming neutrophils start rolling along the blood vessel wall. Rolling is mediated by selectins and modulated by integrins^4^,^5^. Unlike naïve lymphocytes, neutrophils are capable of rolling at high wall shear stress (WSS), *τ* 6. This ability of neutrophils is closely related with the (i) catch-bond behavior of selectins, (ii) deformability of neutrophils, which reduces the hydrodynamic drag and increases the area of contact with the wall, (iii) formation of tethers that bear the loads of force and torque^7^, and (iv) formation of slings that can serve as self-adhesive substrates^8^.

Tethers are long sub-micron diameter tubes pulled out from neutrophils rolling under hydrodynamic drag. Tethers form behind neutrophils, originating from microvilli, and bind to the vessel wall through P-selectin glycoprotein ligand -1 (PSGL-1) expressed on the tips of the microvilli binding to selectins^8^. From *in vitro* experiments, tether tension force is estimated to reach ~80 pN^6^, such that a single tether is expected to significantly contribute to the forces resisting the hydrodynamic drag on the neutrophil (~470 pN at 10 dyn/cm^2^). Mechanical properties of the tethers allow them to substantially stretch at largely unchanged tension force as the neutrophil rolls forward^7^. Eventually the bonds that anchor the tether to the substrate break under the pull of the rolling neutrophil, which we call tether breaking.

Tethers were first identified with DIC microscopy in flow chamber experiments^9^,^10^ behind neutrophils rolling on functionalized glass substrates. Tethers of neutrophils with fluorescently labeled membrane were imaged using TIRF microscopy with the quantitative dynamic foot printing (qDF) method^6^,^11^. In the high resolution qDF images, the tether anchoring points appeared as bright dots up to 16 μm behind the rolling cell, suggesting that tethers can be at least 16 μm long. The same technique was used to discover slings, up to 22 μm long structures found on the substrate in front of rolling neutrophils. However, the TIRF microscopy imaging did not allow observing the tether-to-sling transition directly. Here, we tested the hypothesis that slings originate from tethers that swing around the rolling cell after detaching from the selectin substrate.

For a neutrophil rolling on a horizontal substrate on a regular vertical (upright or inverted) microscope, capturing an entire tether or sling is difficult, because these structures have large extensions along the vertical (Z-) axis, whereas the imaging is always in a horizontal (XY-) plane. Confocal Z-axis optical sectioning has low temporal and spatial resolution^12^ and may also induce photo-bleaching and photo-toxicity. Therefore, it would be a poor match for the fast dynamic process of neutrophil rolling.

Side view flow chambers were previously implemented using 45° tilted mirrors^13^,^14^,^15^ or a horizontally mounted objective^16^. In another approach, rolling cells were imaged on a vertical wall of an ~0.6 mm round capillary with agarose walls^17^, revealing WSS-dependent deformation of human neutrophils. These setups did not allow imaging with high-resolution (high numerical aperture), low working-distance objectives. Our new side view flow chamber can be interrogated with high numerical aperture immersion objectives for maximum spatial resolution.

Here we use a microfluidic flow chamber to study primary mouse neutrophils rolling on a vertical (XZ-plane) wall, such that entire tethers and slings are observed at high resolution in a single XY-plane with high numerical aperture objectives. This setup enables the first direct observation of tether-to-sling transition on well-defined substrate at physiologically relevant WSS values.

## Methods

### Side view flow chamber

**Figure 1:**
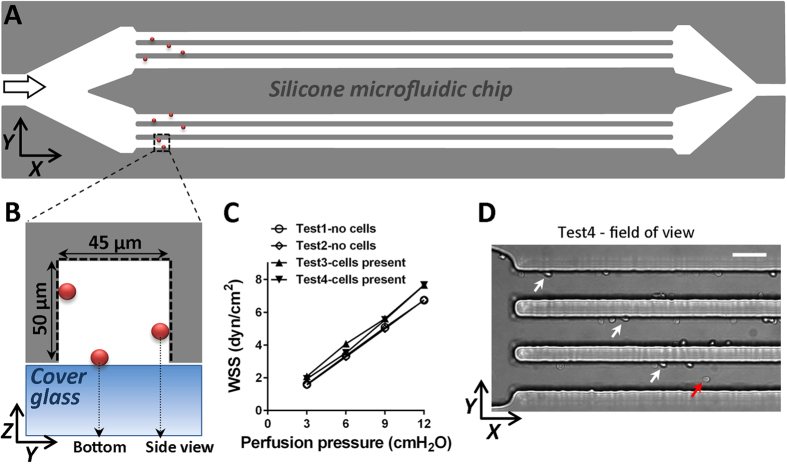
Side view flow chamber. The flow chamber is assembled by vacuum clamping a silicone (PDMS) microfluidic chip against a #1.5 cover glass. (**A)** Schematic of the flow chamber; large arrow indicates flow direction; rolling cells are symbolized by (not to scale) red dots. (**B)** Cross section of one test channel in Y-Z plane. For side and bottom view, cells rolling on the cover glass bottom and lateral silicone wall, respectively, are imaged. (**C)** Calibration of wall shear stress (WSS) as a function of perfusion pressure with and without cells. (**D)** Low magnification (20x) brightfield image of rolling leukocytes in the side view flow chamber. White arrows indicate the cells visible from side view and red arrow indicates a cell visible from bottom view perspective. Scale bar represents 45 μm.

The microfluidic device consisted of a polydimethylsiloxane (PDMS) chip with micro-channels engraved on its surface and a #1.5 cover glass sealing the micro-channels (Fig. 1). The chips were cast using a silicon wafer with a lithographically fabricated relief of a negative photoresist (SU8-2000 by Microchem, Westborough, USA) on its surface as a master mold^18^. The surface of PDMS was functionalized with reactive amino groups using silane chemistry. The microfluidic device had one inlet, one outlet, and 12 identical parallel perfusion channels with width *w* = 45 μm, height *h *= 50 μm, and length of 5 mm (Fig. 1A,B). In experiments with washed neutrophils, the neutrophil suspension and buffer were held in reservoirs connected to the device inlet and outlet, respectively, through PE10 (Becton Dickinson, New Jersey, USA) tubing. The perfusion was driven by a positive differential pressure, ΔP, between the inlet and outlet that was applied hydrostatically by placing the inlet reservoir above the outlet reservoir. The channels were first primed with PBS, then functionalized via perfusion with 1 μg/ml P-selectin-Fc (R&D Systems Inc., Minneapolis, USA) PBS solution for 15 min, and finally blocked with casein in PBS blocking solution (Thermo Fisher Scientific, Waltham, USA) for 1 h. P-selectin – PSGL-1 interaction dependence of rolling was tested via adding P-selectin (clone RB40.34, 5 μg/ml for 15 min) or PSGL-1 (clone 4RB12, 5 μg/ml for 15 min) blocking monoclonal antibody to the cell suspension. The monoclonal blocking antibodies were purified from hybridoma supernatant at the biomolecular facility of the University of Virginia (Charlottesville, USA). The coefficient of proportionality between ΔP and WSS in the perfusion channels was determined by perfusing the device with an aqueous suspension of 500 nm fluorescent beads (Polysciences, Warrington, USA) and calculating their maximal velocity *v*_max_ vs. ΔP. The value of *v*_max_ was calculated by dividing the length of the streak lines of the beads near the axis of symmetry of the channel by the exposure time, and WSS at the mid-plane of the channel (25 μm from the bottom), *τ*, was calculated as *τ* = 4.3*ηυ*_*max*_/*w*, where *η* is the viscosity and 4.3 is a factor derived by numerical simulation.

### Neutrophil preparation

The experimental animal procedures of this study were performed in accordance with approved guidelines. These guidelines were approved by the Institutional Animal Care and Use Committee of La Jolla Institute for Allergy and Immunology, which is an AAALAC international accredited facility. For each experiment, bone marrow from a wild type female mouse was collected, resuspended in PBS and filtered through a 40 μm nylon cell strainer (Biologix, Lenexa, USA). From that sample, neutrophils were enriched at room temperature via negative selection with a customized neutrophil enrichment kit (StemCell Technologies, Vancouver, Canada). FACS measurements indicated that >70% of cells were neutrophils (Ly-6G^+^), and the major contaminating cells were monocytes. The neutrophil isolate was resuspended at 2.5 million cells/ml in RPMI supplemented with 10% fetal calf serum (Gemini, West Sacramento, USA) and 0.1% penicillin-streptomycin (Gibco – Thermo Fisher Scientific, Waltham, USA). To label the cell membrane, 4 μm Vybrant-DiO (Invitrogen, Carlsbad, USA) or 1 μm Cell Mask Green (Invitrogen, Carlsbad, USA), or 5 ng/μl (1:100) anti Ly-6G antibody conjugated with Alexa Fluor 488 (clone RB6–8C5, eBioscinece, San Diego, USA) was added to the cell suspension. After 10 min incubation, with washing (DiO) or without washing (Cell Mask Green, Ly-6G-AF488), the suspension was perfused through the flow channels. As soon as rolling cells were visible, the perfusion solution was switched to PBS to reduce the background signal by washing out the solution containing free dye.

### Imaging and data analysis

Fluorescence imaging of rolling neutrophils was performed with a Leica SP5 confocal microscope (Leica Microsystems Inc., Buffalo Grove, USA) using a 63× 1.3 glycerol immersion objective. For bottom view images, cells rolling on the bottom surface were imaged, while for side view, cells rolling on the side walls were imaged (Fig. 1B). A resonant scanner was used to record rolling neutrophils at 10 frames/second; a conventional scanner was used to image Z-stacks of photofixed cells. For photofixation, the epifluorescence light source was turned on for 5 seconds, which resulted in intermittent or permanent cell arrest without visible damage to the cell. DIC imaging was done with Zeiss LSM 780 microscope (Carl Zeiss Microscopy, Thornwood, USA). Basic image processing and distance measurements were performed with Fiji image analysis freeware (website: fiji.sc). Z-stack reconstruction and cell tracking was done with Imaris image analysis software (Bitplane, Windsor, USA). For statistical analyses Student’s T test or correlation analysis was performed.

## Results

### Flow chamber

Primary mouse bone marrow neutrophils rolled at 5.2 ± 1(SEM) μm/s at WSS = 6–10 dyn/cm^2^ (n = 28) (Supplementary Movie 1). Neutrophil rolling was specific as it was not observed in non-coated channels, channels pre-incubated with P-selectin blocking or PSGL-1 blocking antibody in the cell suspension.

### Tethers observed in bottom view and side view

**Figure 2:**
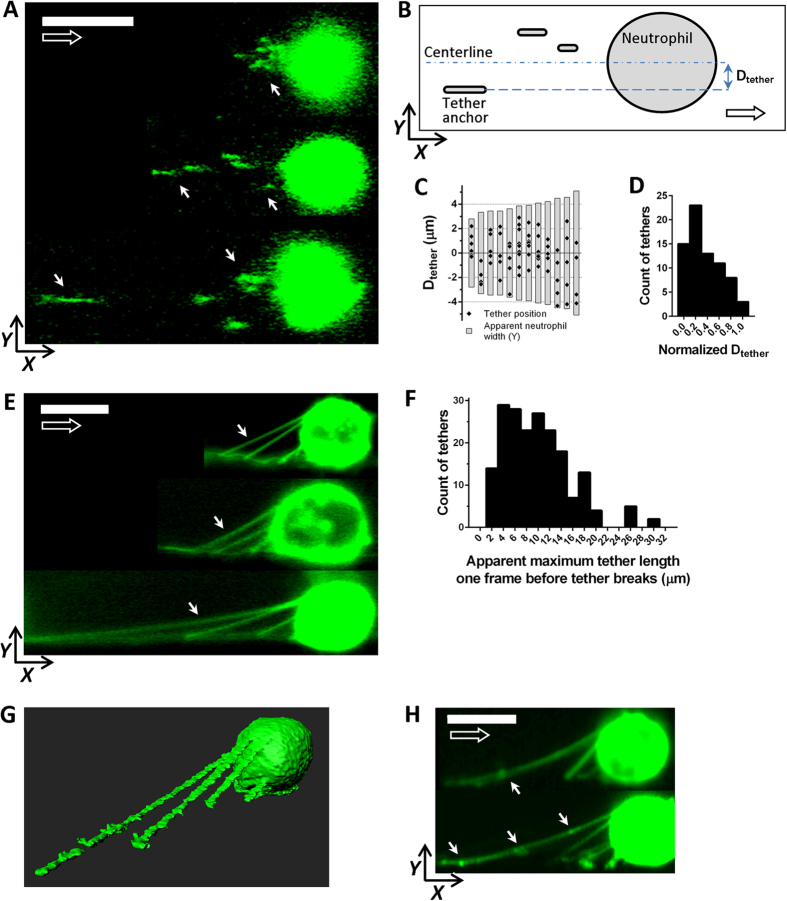
Tethers of rolling/just arrested neutrophils. **(A)** Bottom view confocal micrographs of neutrophils rolling from left to right. The focal plane is just above the cover glass to capture the tether anchoring points, which appear as short lines (some marked by white arrows). (**B)** To quantify tether position along the neutrophil width the distance between neutrophil Y centerline and the Y projection of the tether was measured (D_tether_). (**C)** Each gray bar represents the apparent width of an individual neutrophil from the bottom view imaging. Black diamonds indicate the tether’s D_tether_ values. Whereas the positions of tethers are widely distributed, their number is maximal in the central 2 μm section along the Y-axis. (**D)** Histogram of D_tether_ values, (normalized to neutrophil half-width) mean (±SEM) = 0.35 ± 0.03, min = 0.01, max = 0.96, n = 73. (**E)** Side view confocal images of neutrophils taken immediately after their arrest with averaging over several frames to enhance signal-to-background ratio. Only tethers within the 2 μm depth of field are visible. (**F)** Histogram of the apparent tether length, the distance between the tether anchor point and the point where the tether merges with the cell body, as measured in side view immediately before tether breaks; mean (±SEM) = 9.8 ± 0.41 μm, min = 1.2 μm, max = 30.1 μm, n = 193. (**G)** 3D reconstruction image of a recently arrested neutrophil. A neutrophil rolling on the side wall was photo-fixed and a confocal Z-stack was taken. (**H)** In some cases grape-like structures were observed on the tethers (indicated by white arrows). Scale bars are 8 μm and open arrows indicate the flow direction.

Bottom view images of rolling neutrophils showed tether anchor points behind the cell body as expected. Due to the limited optical section thickness, only short segments of tethers were visible (Fig. 2A). Neutrophils rolling at 6–10 dyn/cm^2^ had 4–10 tethers (median value 5, n = 12). The tethers were distributed across most of the apparent neutrophil width (Fig. 2C) with the numbers of tethers declining towards the neutrophil periphery (Fig. 2D).

Side view images taken immediately after neutrophil arrest revealed the whole tether segments between the cell body and the side wall (Fig. 2E). The side view images show many but not all tethers of each neutrophil, because the optical section thickness is about 2 μm, but tethers are distributed across ~7 μm of the cell width as shown by bottom view images (Fig. 2C). Analysis of 193 tether side view images showed that at 6–10 dyn/cm^2^ WSS, the average apparent tether length, determined as the distance between the point where the tether appears to emerge from the cell body and the point where the tether reaches the wall of the flow channel was 9.8 ± 0.4(SEM) μm; the longest tether length was 30.1 μm and the shortest was 1.2 μm (Fig. 2F). The tether length measured in side view was significantly (p < 0.0001) longer than the tether length measured in bottom view (6.5 ± 0.4(SEM) μm, ranging from 1 to 24.8 μm; n = 58), suggesting systematic undersampling of long tethers in bottom view mode.

In some experiments, rolling neutrophils were photofixed to obtain a whole-cell side view Z-stack (Fig. 2G and Supplementary Movie 2). The side view images showed that some tethers have grape-like structures along their length (Fig. 2H). To show that tether formation is not an artefact caused by membrane labeling with membrane intercalating dyes (DiO and Cell Mask Green), non-labeled rolling neutrophils were imaged with DIC microscopy (Supplementary Fig. 1) or rolling neutrophils labeled with Ly-6G-AF488, a fluorescently labeled antibody against a neutrophil surface marker, were imaged with confocal microscopy (Supplementary Fig. 2). Both of these methods showed tethers similar to the ones observed with the DiO and Cell Mask Green dyes. The side view images also confirmed the previously reported elongated shapes of rolling neutrophils under shear stress^19^,^20^.

### Tether break results in micro jump

**Figure 3:**
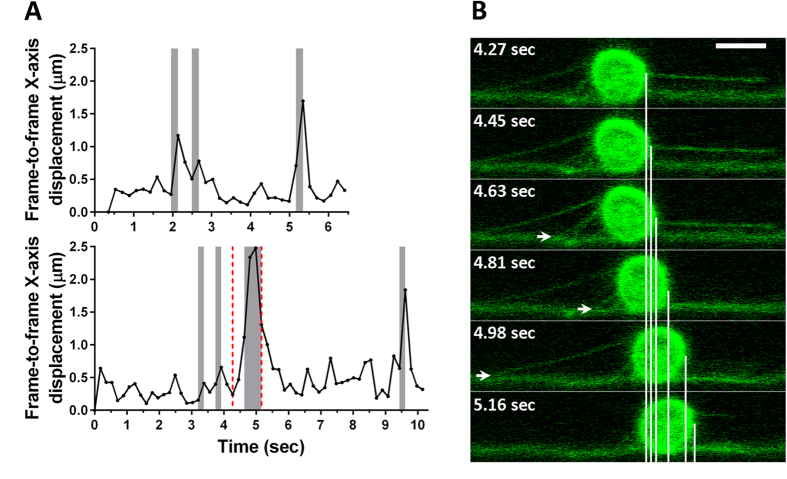
Tether break results in micro-jump. (**A)** The position of the center of mass of rolling neutrophils was tracked from video recordings as a function of time. The last frames where a certain tether is visible and the first frames where the tether is not visible any more are highlighted by grey columns, which are interpreted as time points of tether break events. Tether breaks are found to concur with greater cell displacements over the fixed time interval between the frames, indicating significant short-term accelerations or micro-jumps. (**B)** Six consecutive frames from Cell 2 sequence (range indicated by red dashed line on panel A) are shown. White arrows indicate tether anchoring points on the last frame where the tether is visible. The vertical lines indicate the front of the cell at different time points. Two short tether breaks precede a long tether break, with each break resulting in a micro-jump. The neck of the sling in front of the cell rotates clockwise with the cell, approaching the substrate (side wall) in the process. Scale bar is 8 μm.

Next, we asked what role tethers played in neutrophil rolling. If individual tethers bear significant loads, the neutrophil rolling should significantly accelerate immediately after breaking of a tether. Frame-to-frame neutrophil displacement was analyzed between the last frame where an intact tether was seen (frame T) and the two following frames (frames T + 1 and T + 2). The displacement between T and T + 1 was 1.7 ± 0.1(SEM) times greater than between T + 1 and T + 2 (n = 22), indicating that a rolling neutrophil greatly accelerates, effectively making a jump, immediately after a tether breaks. In some rolling neutrophils, multiple consecutive tether breaks could be observed (Fig. 3A), and large neutrophil displacements (jumps) immediately followed. In one cell (cell 2 in Fig. 3A,B), multiple tethers broke within ~0.5 second, resulting in a very large jump. These findings suggest that individual tethers make significant contributions to the resistance force preventing the cell from moving with the flow.

### Tether-to-sling transition

**Figure 4:**
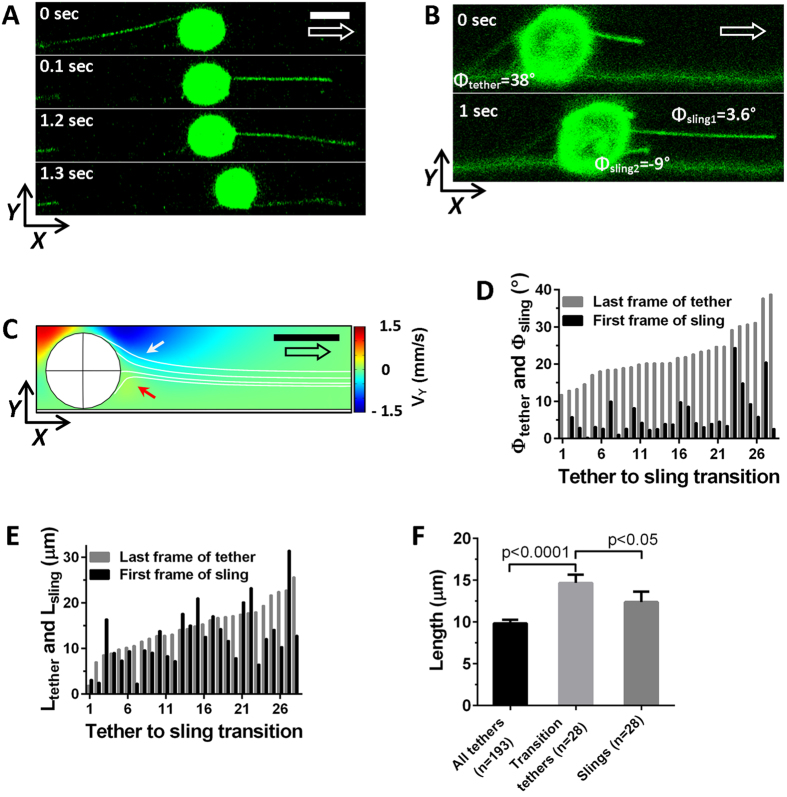
Tether-to-sling transition. (**A)** Key frames of a tether-to-sling transition from a side view record. Transition happens within 100 ms, but it takes 1.2 sec until this sling reaches the substrate. During that period the sling would not be visible from the bottom view perspective. (**B)** Side view images allowed measuring the angle between the bottom surface and tethers (Φ_tether_) or slings (Φ_sling_). Two consecutive frames with a tether-to-sling transition are shown. (**C)** Computer fluid dynamic simulation of flow conditions around the neutrophil at 10 dyn/cm^2^ wall shear stress. The heat map indicates the direction and magnitude of the flow component perpendicular to the bottom surface (V_Y_). At the right upper quadrant of the cell V_Y_ points down (indicated by white arrow) and at the right lower quadrant of the cell V_Y_ points up (indicated by red arrow). The four white lines originating on the right side of the cell indicate how the slings would align under the modeled stream conditions. (**D)** Last tether/first sling angle pairs were measured on tether-to-sling transitions. The angle pairs are shown as bar-doublets, where the gray bar represents tether angle and the black bar represents sling angle. Mean (±SEM) last tether angle = 22.3 ± 1.2°, min = 11.8°, max = 38.7°. Mean (±SEM) first sling angle = 6 ± 1°, min = 0°, max = 24.3°. (**E)** Final lengths of individual tethers (grey bars, min = 1.8 μm, max = 25.6 μm) and initial lengths of slings that originated from those tethers (black bars, min = 2.3 μm, max = 31.5 μm) for different tether-to-sling transitions. (**F)** Average (±SEM) lengths of final lengths of all tethers, tethers before tether-to-sling transitions (Transition tethers), and initial lengths of slings. Scale bars indicate 8 μm, large arrows indicates flow direction.

This side-view flow chamber allows “for the first time” to visualize the formation of slings. Specifically, we tested the hypothesis that slings derived from tethers, which required detailed observation of the formation of tethers and slings. In side view (Fig. 4A and Supplementary Movie 3) and bottom view (Supplementary Fig. 1 and Supplementary Movie 4) recordings, slings were observed to attach to the substrate in front of the cell after tether breaks, suggestive of a tether-to-sling transition taking place. However, considerable delay (3.2 ± 0.8(SEM) sec) between the two events made the evidence for the tether-to-sling transition from the bottom view chamber experiments less convincing and also made it difficult to establish which specific tether became the sling. Unlike the bottom view chamber, where a sling only becomes visible after it attaches to the substrate, the side view enabled observing slings in frames immediately following those with tether breaks, within ~100 ms of their formation and long before they could be seen in the bottom view. During that time interval, slings could be seen stretched along the stream lines in front of the cell (Fig. 4B). Computational fluid dynamics (CFD) showing the Y velocity map around the rolling cell confirmed that initially, slings experience a velocity toward the wall (Fig. 4C). As the cell continued rolling, bringing the point of attachment of the sling to the cell closer to the substrate, the stretched sling got closer to the substrate as well. The side view time series indicated that about 15% of all breaking tethers (28 of 193) formed slings. Slings were also observed on Ly-6G-AF488 labeled rolling neutrophils (Supplementary Fig. 2), supporting that sling formation is not an artefact caused by membrane labeling with DiO or Cell Mask Green.

We measured the angle between the tether and bottom surface (Φ_tether_). Consistent with the length of the tethers and the diameter of the neutrophil, the average Φ_tether_ was 22.3 ± 1.3(SEM)° (Fig. 4D), ranging from 12° to 38°. The angle between the sling and a line parallel to the wall averaged 6 ± 1(SEM)° (Φ_sling_) (Fig. 4B,D). CFD showed that the streamlines in front of the rolling cell were tilted downwards. However, as the cell rotated and the sling approached the substrate, CFD predicted an upward angle (Fig. 4C). This was experimentally confirmed by looking at short slings near the wall (Fig. 4B, lower panel).

We compared the lengths of tethers immediately before and slings immediately after the tether-to-sling transitions (Fig. 4E). The average final tether length was 14.6 ± 1(SEM) μm, significantly shorter (p < 0.05) than the average initial sling length of 12.3 ± 1(SEM) μm. The sling-forming tethers were significantly (p < 0.0001) longer (14.6 μm) than the average (9.8 ± 0.41(SEM) μm) (Fig. 4F), suggesting that slings preferentially form from longer tethers. On average, the slings were 81 ± 7% of the length of the tethers they originated from.

## Discussion

Here, we introduced a new microfluidic side-view flow chamber compatible with high resolution microscopy that enabled the first direct observation of the tether-to-sling transition, a process of fundamental importance for leukocyte adhesion under high shear stress. In previous studies, tethers were hypothesized to form slings [6], but there was no direct evidence for that. Tether breaks could not be reliably matched to sling formation, because slings are invisible under TIRF microscopy until they are very close to the substrate.

Our data indicate that about 15% of all tethers form slings. This might be an underestimate, because the side view flow chamber records only a ~2 μm section in the XZ plane and thus might miss slings that may have moved out from the focal plane in the Y direction. This can be caused by random movement of rolling neutrophils in the Y direction, which is expected whenever the key anchor point is off-center^21^,^22^. When a tether breaks and another off-center tether becomes load-bearing, the cell will pivot about the Y-axis until the cell center is downstream from the new tether. Since the sling cannot provide any mechanical stability while detached (before reaching the wall), the sling will passively swing in the Y direction, forced by the Y movement of its neck where it is attached to the rolling cell^23^,^24^,^25^.

The transition from a tether behind a cell to a sling in front of the cell is completed within less than one frame (100 ms). In the 28 tether-to-sling transitions, we could only see slings on the substrate or aligned along the flow streamlines in front of rolling cells and never above the rolling cell. This observation is consistent with the expected physical properties of slings, sub-micron diameter tubes with low resistance to bending^7^,^26^,^27^,^28^ that can only be stable (as needed to produce sufficiently sharp images under 100 ms exposure) under longitudinal tension, but not under transverse forces. Indeed when slings are in front of rolling cells, before landing on the substrate, they are closely aligned along the streamlines.

On most of the side view transition records, the sling can be reliably matched to a broken tether. Slings spend several hundred milliseconds “hanging” in the fluid stream before landing. This explains the delay between tether break and sling formation observed with qDF. The hanging slings enclose an average angle of 6 ± 1(SEM)° with the bottom surface. This is consistent with the notion that the slings follow the flow profile in front of the cell and a previous intravital microscopy study with particle tracking velocimetry^29^. The floating sling does not reach the wall until the rolling neutrophil has rotated far enough so the origin (“neck”) of the sling is near the wall. This observation is consistent with slings being anchored to the cytoskeleton^30^,^31^, but the nature of this anchorage is currently unknown and awaits further research.

The side view chamber enables more accurate measurements of the tether lengths as compared with the bottom view chamber. However, the apparent lengths of the tethers in the side view flow chamber are still underestimates, because a segment of the tether close to its neck will be obstructed by the brighter cell, when the neck is not in the equatorial plane of the cell. If a tether originates from the cell body in a plane different from the equatorial plane, the part of the tether “behind” or “in front of” the cell equator is invisible and will not be measured. This is significant as shown in (Fig. 2), where many tethers originate in planes other than the equatorial plane. Thus, a complete understanding of rolling with tethers and slings is only possible through side AND bottom (or top) view imaging as reported here.

The side view images showed that some tethers have grape-like structures along their length, indicating that the grapes observed by electron microscopy of fixed neutrophils with tethers^6^ are not fixation artefacts. Similar grape-like structures were observed on tethers of rolling platelets^32^. The nature of these structures is unknown; one theory is that these are remnants of microvilli which are pulled from the cell with the membrane into the tether.

We observed that each break of a tether results in a major short-term acceleration of the cell rolling (a microjump). This indicates that, immediately before breaking, each tether is load-bearing, significantly contributing to the forces opposing hydrodynamic drag on the cell, corroborating previous reports^6^,^10^.

In its current configuration, the side view flow chamber can show many rolling cells from perfusion with 250 μl cell suspension containing 500,000 cells. The volume of cell suspension required can be further reduced in a device with a smaller number of perfusion channels. The PDMS walls can be readily coated with proteins thus making the side view flow chamber useful for studies of neutrophil arrest and interaction of other types of leukocytes^33^,^34^ or platelets^35^,^36^.

Here we studied neutrophils rolling on P-selectin with no integrin ligand. Tether and sling formation on more complex substrates has not been studied. Neutrophils rolling on E-selectin also form slings and tethers^6^. Little is known about tether and sling formation of other leukocytes, except that T-helper 1 (Th1) CD4 lymphocytes but not naïve CD4 T lymphocytes rolling on P-selectin form slings and tethers^6^.

In summary, we directly show on neutrophils rolling on P-selectin that some tethers form slings that are aligned along streamlines in front of the cell before landing on the substrate; tethers originate from almost anywhere across the leukocyte width; tethers are load-bearing and thus directly relevant for cell rolling. We conclude that the new side and bottom view flow chamber is a versatile tool for studying cell rolling, adhesion and migration under flow.

## Additional Information

**How to cite this article**: Marki, A. *et al.* Microfluidics-based side view flow chamber reveals tether-to-sling transition in rolling neutrophils. *Sci. Rep.* **6**, 28870; doi: 10.1038/srep28870 (2016).

## Supplementary Material

Supplementary Information

Supplementary Movie 1

Supplementary Movie 2

Supplementary Movie 3

Supplementary Movie 4
