# Supplementary material for: Microfluidics-based side view flow chamber reveals tether-to-sling transition in rolling neutrophils
Source: Sci Rep. 2016 Jun 30;6:28870. doi: 10.1038/srep28870 (PMC4928115; doi:10.1038/srep28870)
Supplement: Supplementary Information [file srep28870-s1.pdf]

Supplementary information

Microfluidics-based side view flow chamber reveals tether-to-sling transition in rolling neutrophils

Alex Marki [1], Edgar Guiterrez [2], Zbigniew Mikulski [1], Alex Groisman [2], Klaus Ley [1,3]

[1] La Jolla Institute for Allergy and Immunology, La Jolla, CA

Department of [2]Physics and [3]Bioengineering, University of California San Diego, La Jolla, CA

Correspondence:

Klaus Ley, M.D.
Professor and Head
Division of Inflammation Biology
La Jolla Institute for Allergy & Immunology
9420 Athena Circle Drive
La Jolla, CA 92037
(858) 752-6661 (tel)
(858) 752-6985 (fax)
klaus@liai.org

Supplementary Figure:

1. Bottom view and side view of tethers and slings labeled with Alexa Fluor 488 conjugated Ly-6G

Top, multiple tether anchoring points are visible from bottom and side view. Bottom, a tether and an adhered sling are visible in side view. Scale bars indicate 8 µm, arrows indicate rolling direction.

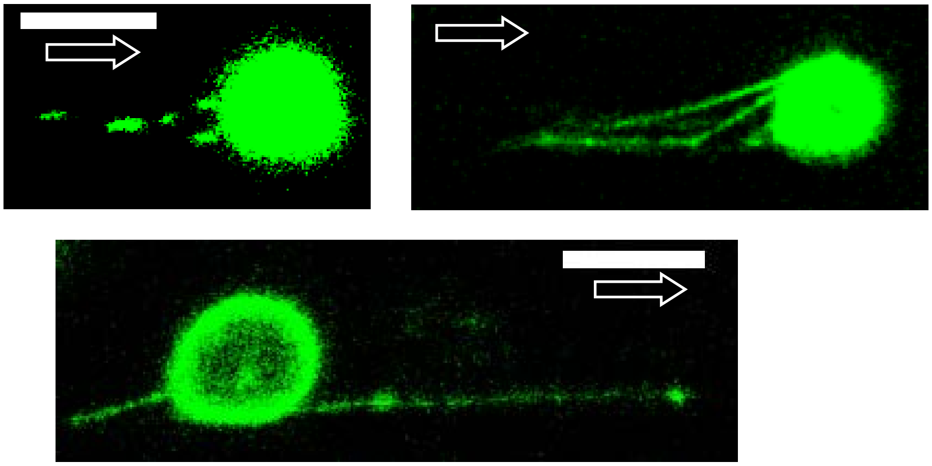

2. Side view of a non-labeled neutrophil tether imaged with DIC

A tether with a grape like structure (indicated by the small arrows) is visible behind a rolling neutrophil. Large arrow indicates rolling direction.

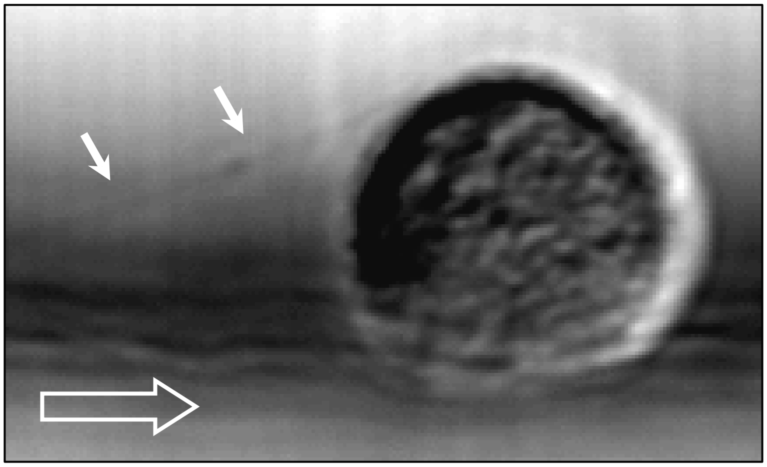

3. Bottom view of tether to sling transition

Tether anchoring point breaks during the first 100 ms of the recording; however, the slings become visible only about 400 ms later as a blurred image. It takes another 1.8 sec until the slings is clearly visible, i.e. the sling is within about 2 µm of the substrate. Scale bar indicates 8 µm, open arrow shows the flow direction.

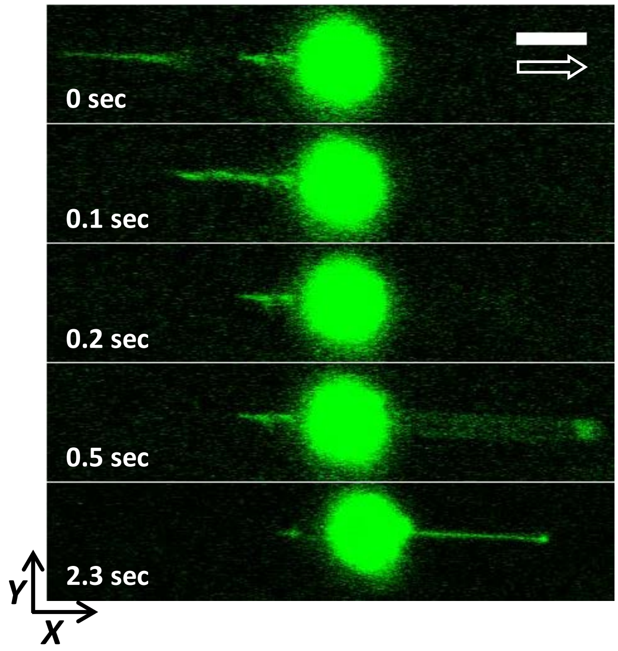

Supplementary movies:

1. Isolated neutrophils rolling in side view flow chamber

Perfusion pressure was set to reach 10 dyn/cm^2 wall shear stress. The sharply visible neutrophils roll on the bottom surface and are visible from bottom view, the blurred neutrophils roll on the lateral wall of the flow chamber and are visible from side view.

2. 3D reconstructed neutrophil with tethers

Arrested neutrophil with four tethers is visible. First half of the movie shows the reconstructed raw data, the second half of the movie shows the model applied on the reconstructed raw data.

3. Tether-to-sling transition in side view

4. Tether-to-sling transition in bottom view
